# Supplementary material for: Trimetazidine Improves Mitochondrial Dysfunction in SOD1G93A Cellular Models of Amyotrophic Lateral Sclerosis through Autophagy Activation
Source: Int J Mol Sci. 2024 Mar 13;25(6):3251. doi: 10.3390/ijms25063251 (PMC10970050; doi:10.3390/ijms25063251)
Supplement: Supplementary file 1 [file ijms-25-03251-s001.zip › ijms-2864874-supplementary.pdf]

Molecular Neurobiology

Trimetazidine ameliorates mitochondrial dysfunction in Amyotrophic Lateral Sclerosis SOD1^{G93A} cell models via autophagy activation.

Illari Salvatori[1,2§], Valentina Nesci[2,3§], Alida Spalloni[2], Veronica Marabitti[4], Maurizio Muzzi[5], Henri Zenuni[6], Silvia Scaricamazza[2], Marco Rosina[2,3], Gianmarco Fenili[2], Mariangela Goglia[6], Laura Boffa[3], Roberto Massa[6], Sandra Moreno[5], Nicola Biagio Mercuri[2,6], Francesca Nazio[4], Patrizia Longone[2], Cristiana Valle[2,7], Alberto Ferri[2,7]

[1] University of Roma "La Sapienza", Department of Experimental Medicine, Rome, Italy
[2] IRCCS, Fondazione Santa Lucia, Rome, Italy
[3] Fondazione PTV Policlinico Tor Vergata, Unit of Neurology, Rome, Italy.
[4] Tor Vergata University of Rome, Department of Biology, Rome, Italy
[5] University of Roma Tre, Department of Science, LIME,Rome, Italy.
[6] Tor Vergata University of Rome, Department of Systems Medicine, Unit of Neurology, Rome, Italy.
[7] National Research Council (CNR), Institute of Translational Pharmacology (IFT), Rome, Italy

§ These authors have equally contributed to this work.

Correspondence to:

Cristiana Valle, National Research Council, Institute of Translational Pharmacology (IFT), Rome, Italy.
Fax: +39 06 501703323, Tel. +39 06 501703087 Email: cristiana.valle@cnr.it

Alberto Ferri, National Research Council, Institute of Translational Pharmacology (IFT), Rome, Italy.
Fax: +39 06 501703323, Tel. +39 06 501703070 Email: alberto.ferri@cnr.it

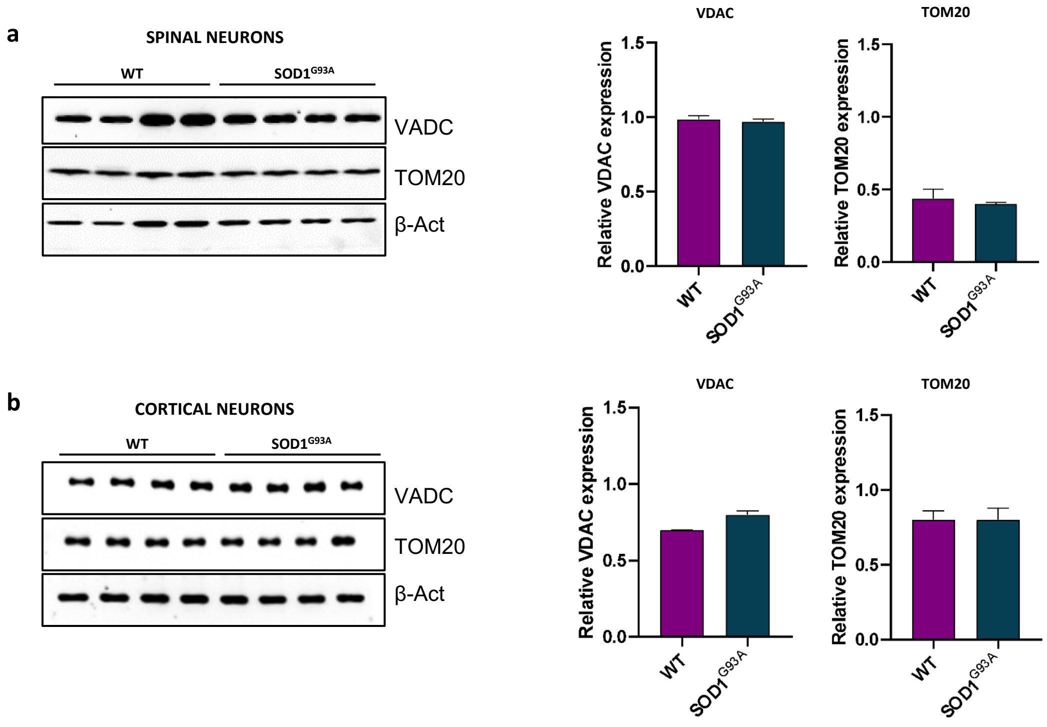

Fig. S1 (a) Representative WB (right) and quantification (left) of VDAC and TOM20 expression in primary spinal neurons wild-type (WT) and SOD1^{G93A} mice. Proteins expression was normalized using β-Actina and represented by arbitrary units. **(b)** Representative WB and quantification as in (a) in primary cortical neurons wild-type (WT) and SOD1^{G93A} mice. Data are presented as mean ± SEM. Statistical analysis was conducted using Student's t test.

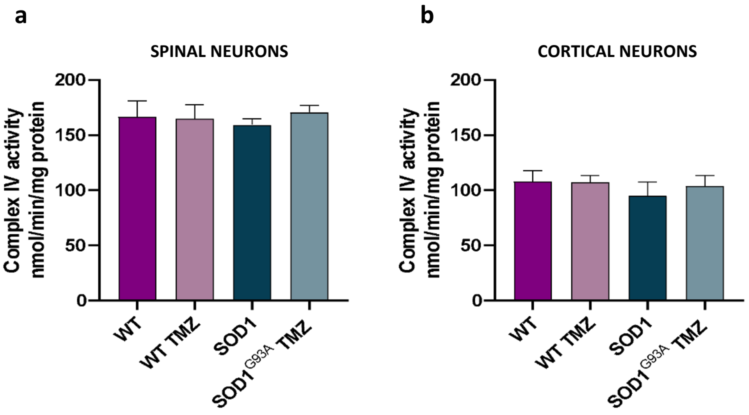

Fig. S2 Representative histograms showing the activity of electron transport chain complex IV in total extract from primary spinal cord **(a)** and cortical **(b)** cell cultures obtained from WT and SOD1^{G93A} mice untreated or treated overnight (ON) with 10μM of Trimetazidine (TMZ). Values are expressed as nmol/min/mg protein, normalised by citrate synthase activity and reported as mean ± S.D. from three independent experiments with each sample in quadruplicate.

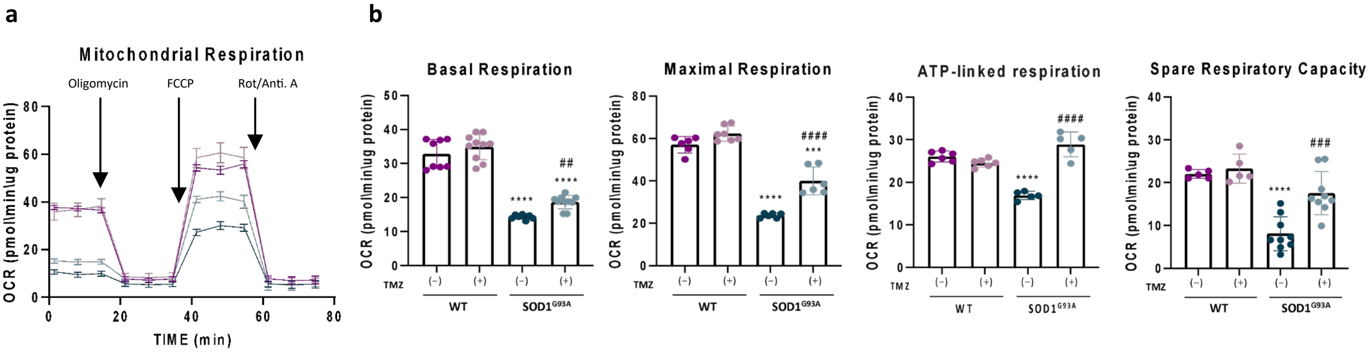

Fig. S3 Trimetazidine treatment restores mitochondrial performances of NSC-34 SOD1^{G93A} cell lines.
(a) Representative profile of measurements of the rate of oxygen consumption ratio (OCR) in NSC-34 cell lines constitutively expressing SOD1 wild type (WT) or mutated (SOD1^{G93A}), untreated or treated with 10 μM TMZ as indicated. **(b)** The histograms show individual parameters for basal respiration, maximal respiration, ATP-linked respiration and spare respiratory capacity, as indicated. Data are presented as means ± SEM, ***$p < 0.001$, ****$p < 0.0001$ compared with wild type; ##$p < 0.01$, ###$p < 0.001$, ####$p < 0.001$ compared with untreated SOD1^{G93A}, n = ≥ 3 per group. p values were obtained using parametric one-way ANOVA with Bonferroni post hoc test.

a

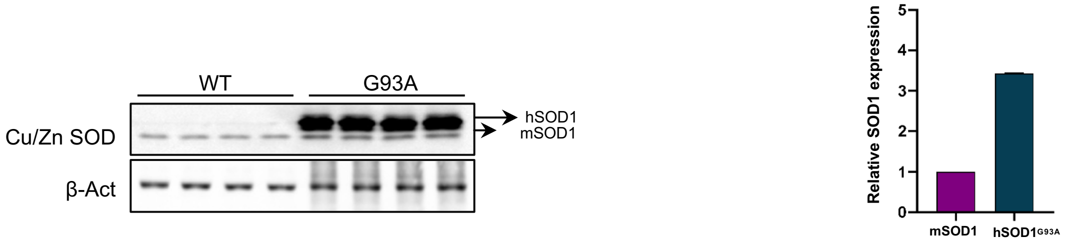

b

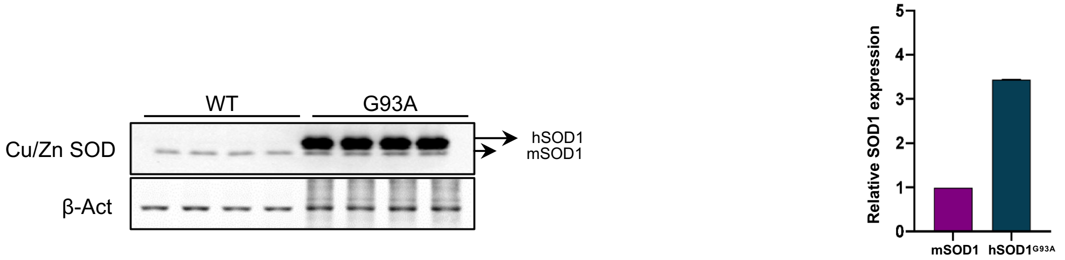

Fig. S4 Representative WB of murine SOD1 (mSOD1) and human SOD1 (hSOD1) expression in primary spinal **(a)** and cortica **(b)** neurons (left) in wild-type (WT) and $SOD1^{G93A}$ (G93A) mice and relative quantification (right) represented by arbitrary units. Data are presented as mean ± SEM. ****$p < 0.0001$ compared with mSOD1. P values were obtained using Student's t test.

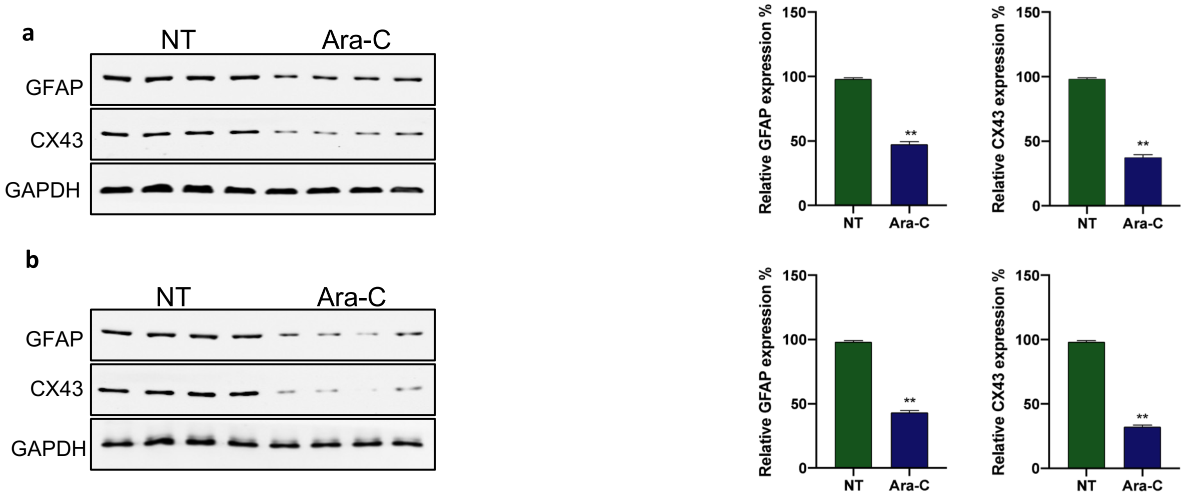

Fig. S5 (a) Representative WB (right) and quantification (left) of GFAP and CX43 in primary spinal neurons untreated (NT) and treated with the astroglial proliferation inhibitor (Ara-C). Proteins expression was normalized using GAPDH. **(b)** Representative WB and quantification as in (a) in primary cortical neurons untreated (NT). Data are presented as mean ± SEM, $^{**}p < 0.01$ compared with NT. P values were obtained using Student's t test.
